# Supplementary material for: Correlates of Calcidiol Deficiency in Adults—Cross-Sectional, Observational, Population-Based Study
Source: Nutrients. 2022 Jan 20;14(3):459. doi: 10.3390/nu14030459 (PMC8838096; doi:10.3390/nu14030459)
Supplement: Supplementary file 1 [file nutrients-14-00459-s001.zip › nutrients-1564555-supplementary.pdf]

SUPPLEMENTARY

MATERIALS

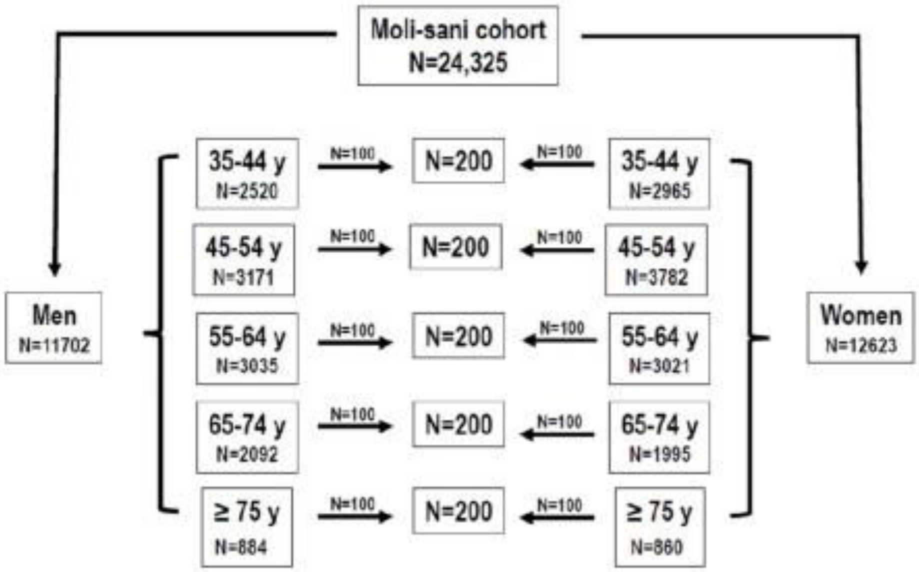

Supplementary Figure S1. Sex and age stratification for selection of target cohort for additional lab test on frozen biological samples.

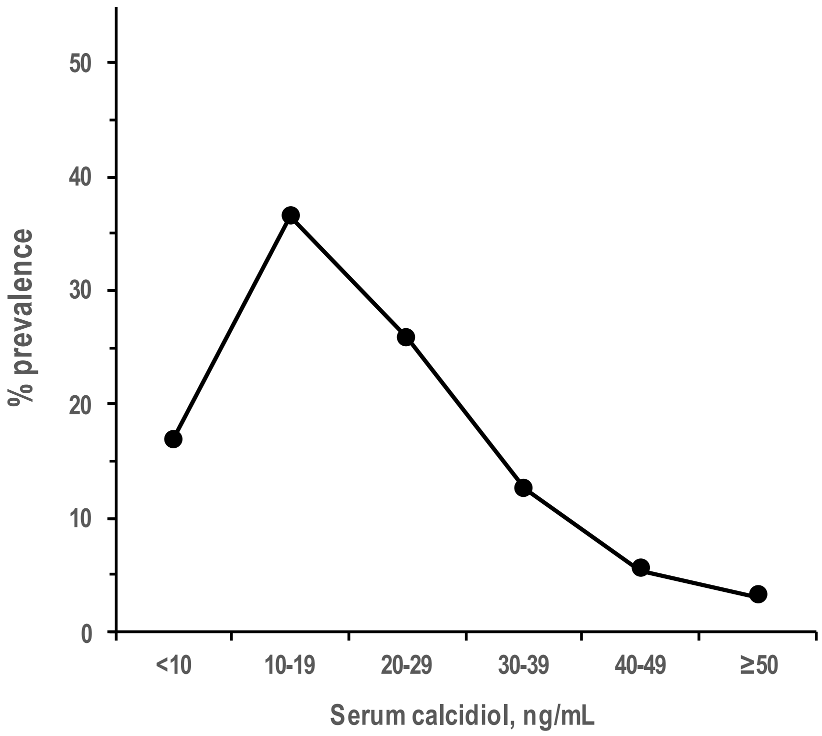

Supplementary Figure S2. Frequency distribution of serum calcidiol (skewness±SE = 1.16±0.08).

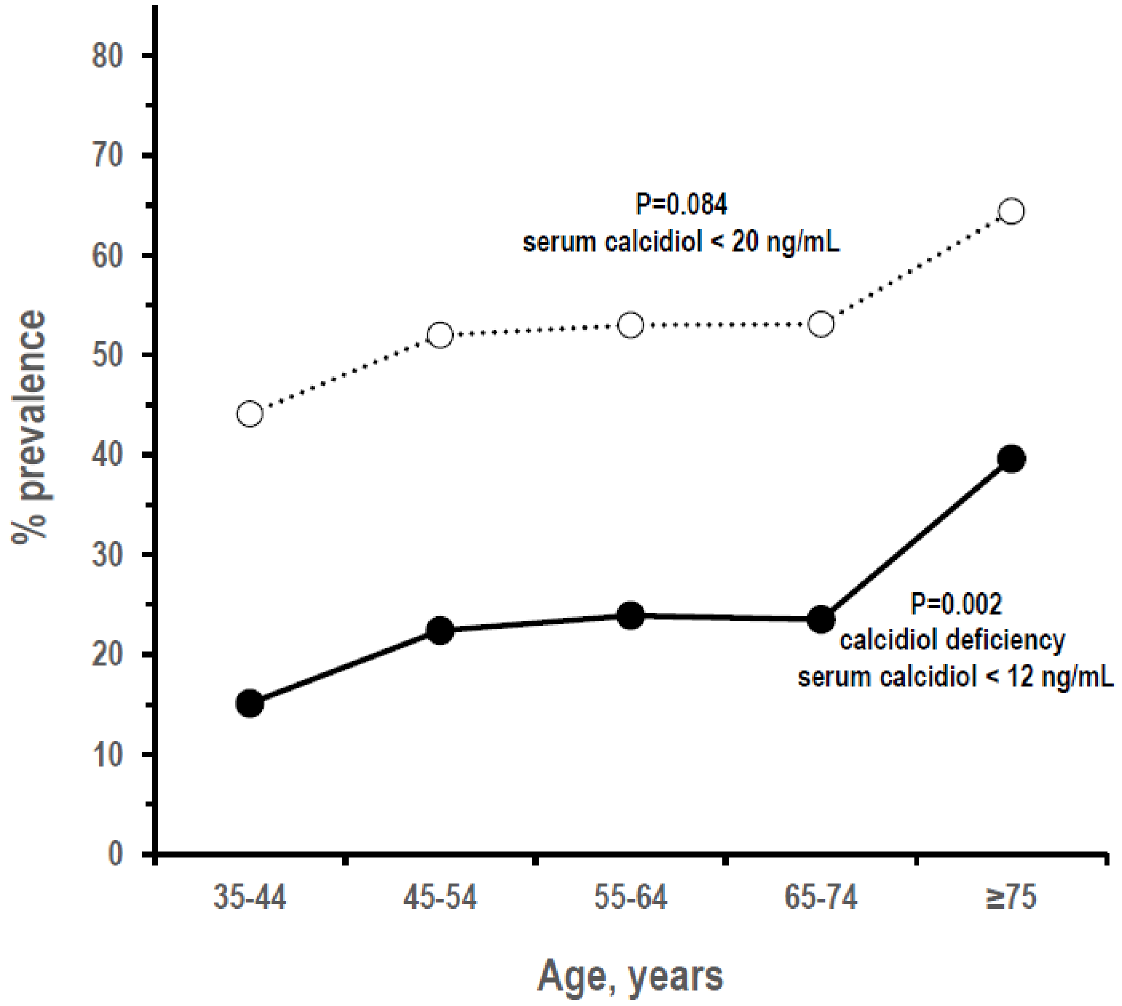

Supplementary Figure S3. Prevalence of calcidiol deficiency (serum calcidiol < 12 ng/mL) and of serum calcidiol < 20 ng/mL by age (P value by chi-square analysis).

Supplementary Table S1. Odds ratio (95%CI) of serum calcidiol < 20 ng/mL in multiple-variable logistic regression.

Independent variables	Reference Interval	Odds ratio (95%CI) of serum calcidiol < 20 ng/mL
Sex	Women vs men	0.69 (0.23/2.04)
Age, years	+1SD	1.21 (0.93/1.56)
High education	No vs yes	0.83 (0.61/1.12)
Daily solar irradiance, MJ/m^2	−1SD	**1.59 (1.38/1.83)**
Leisure physical activity, log MET-h/d	−1SD	**1.27 (1.09/1.47)**
Urinary creatinine, g/24-hour	+1SD	1.49 (0.80/2.77)
Body mass index, kg/m^2	+1SD	1.21 (0.87/1.69)
Waist/hip ratio	+1SD	**1.32 (1.12/1.56)**
Diabetes	Yes vs no	1.08 (0.69/1.69)
eGFR, mL/min x 1.73 m^2	+1SD	**1.29 (1.08/1.55)**
Urinary albumin/creatinine ratio, log mg/g	+1SD	1.00 (0.86/1.15)
Systolic pressure, mm Hg	+1SD	**1.32 (1.07/1.64)**
Diastolic pressure, mm Hg	+1SD	0.91 (0.75/1.09)
Serum total cholesterol, mg/dL	+1SD	**1.22 (1.05/1.41)**
Smoking	Yes vs no	1.20 (0.85/1.69)
Alcohol intake, log g/d	−1SD	**1.13 (1.01/1.26)**
Dietary calorie, kcal/d	+1SD	0.96 (0.80/1.16)
Dietary vitamin D, log IU/d	−1SD	1.00 (0.85/1.19)
Vitamin D supplement	No vs yes	**11.98 (4.05/35.41)**

eGFR = estimated glomerular filtration rate

Bold character for statistically significant odds ratio (95%CI not including one).
